# Time-Resolved Study of Site-Specific Corrosion in a Single Crystalline Silver Nanoparticle

**DOI:** 10.1186/s11671-019-3077-9

**Published:** 2019-07-17

**Authors:** Steffen Trautmann, André Dathe, Andrea Csáki, Matthias Thiele, Robert Müller, Wolfgang Fritzsche, Ondrej Stranik

**Affiliations:** Leibniz Institute of Photonic Technology (IPHT) Jena, Member of the Leibniz Research Alliance - Leibniz Health Technologies, Albert-Einstein-Straße 9, 07745 Jena, Germany

**Keywords:** Corrosion, Nanoparticles, Optical spectroscopy, Atomic force microscopy

## Abstract

We followed over 24 h a corrosion process in monocrystalline triangular-shaped nanoparticles at a single-particle level by atomic force microscopy and optical spectroscopy techniques under ambient laboratory conditions. The triangular-shaped form of the particles was selected, because the crystallographic orientation of the particles is well defined upon their deposition on a substrate. We observed that the particles already start to alter within this time frame. Surprisingly, the corrosion starts predominantly from the tips of the particles and it creates within few hours large protrusions, which strongly suppress the plasmon character of the particles. These observations support the crystallographic model of these particles consisting of a high-defect hexagonal closed packed layer, and they could help material scientists to design more stable silver nanoparticles. Moreover, this described technique can be used to reveal kinetics of the corrosion in the nanoscale of other materials.

**Electronic supplementary material:**

The online version of this article (10.1186/s11671-019-3077-9) contains supplementary material, which is available to authorized users.

## Introduction

Nanoscale metal particles are very interesting objects due to their strong interaction with light [1, 2]. This effect is called localized surface plasmon resonances (LSPR), and its origin is in the oscillation of the particles’ electrons induced by light. Such particles show a high absorption, high scattering efficiency, and strong nanofocusing of the irradiated light. This is the base for a use of these particles as sensor [3], optical label [4], color printing [5], and local heating [6, 7], in different enhancement techniques like fluorescence [8, 9], Raman spectroscopy (SERS) [10], or for photo-catalysis [11, 12]. The performance of many of these effects can be boosted by diverging from the basic spherical shape of the particles to more anisotropic particles [13]. Gold is the most commonly used material for the plasmonic particles due to its relatively good optical properties and excellent chemical stability [14]. However, silver exhibits better optical properties due to its small intrinsic damping, but its disadvantage is in its lower chemical stability. Therefore, it is important to investigate the alteration of the silver nanoparticles in order to improve their stability, which in turn allows wider use of them.

Although investigations on oxidation [15] or corrosion of silver (in the frame of our paper the term “corrosion” covers all reactions Ag(0) ➔ Ag1+ independent from the product such as oxide and sulfide) can be found in the literature, they are preferentially dealing with electrochemical processes on larger electrodes or silver layers [16], but rarely on individual nanoscale particles [17]. Silver tends to tarnish induced by H_2_S, SO_2_, NO_2_, and Cl_2_ [18]. These and other nucleophilic complexing agents catalyze the oxidation of silver [19]. The resulting oxidative etching can lead to changes in optical behavior of silver nanostructures. Chemically induced sulfidizing on edges of silver triangles in solution leads to a redshift in their spectra [20]. In addition to this corrosion mechanism, silver nanoparticles show a strong oxidation in the presence of pure oxygen [21, 22]. Also, electrochemically induced oxidation can initiate morphological changes of triangular-shaped silver polycrystalline particles [23]. In this article, we investigated the chemical stability of single-crystalline silver triangular-shaped nanoparticles (TrNPs) and implications of possible changes on their excellent plasmonic properties [24, 25]. TrNPs can be elegantly synthesized in a solution by wet chemical approaches [26, 27]. We deposited the TrNPs from solution on substrates and stored them in ambient conditions. The anisotropic shape of the TrNPs causes the TrNPs to attach on a surface with their large side, and therefore, their crystallographic orientation is well determined. This is not easily possible to achieve with isotropic particles such as spheres. We were able to observe the alteration process over hours on a single-particle level by monitoring morphological and optical changes by using atomic force microscopy and micro-spectroscopy, respectively. The article describes the results of this observation and suggests a model explaining the observations.

## Materials and Methods

### Synthesis and Immobilization of Silver Triangles

Silver triangles were synthesized following a colloidal two-step method [26], using a microfluidic setup for the seed production [28]. The resulting particles with dimensions of 80–100 nm × 8 nm (edge length × height) exhibit a LSPR of ~ 710 nm. The solutions of the nanoparticles were stored in darkness and argon atmosphere at 4 °C. For the investigation on air, the particles were immobilized on glass substrates with chromium patterns (photolithographic structures for particle localization) by silane chemistry [29]. Therefore, 1% pre-hydrolyzed 3-aminopropyl-triethoxysilane (APTES, Sigma-Aldrich Chemie GmbH Munich, Germany) were incubated at room temperature for 10 min and subsequently washed with water. Ten microliters of the 1:10 diluted particle solution (~ 3.1 × 10^10^ P/ml) were incubated for 10 min on a platform shaker (Vi-bramax 100, Heidolph Instruments GmbH & Co.KG, Schwabach, Germany) at 300 rpm, washed again, and dried. The dried samples were immediately transferred for characterization.

### Characterization of the Morphology and Optical Properties

Morphological characterization of the triangles was done by scanning force microscopy (AFM). Therefore, a Nanoscope IIIa with Dimension 3100 (DI, Santa Barbara, CA USA) was used on air in tapping mode with silicon standard tips (Tap300, Budget Sensors). For single AFM, data evaluation software Gwyddion 2.28 (Czech Metrology Institute, Brno, Czech Republic) was used. For the evaluation of the time series of AFM data, self-written software in Matlab using DipImage library was used. The nanoparticles were characterized by TEM (HR-TEM, JEM 3010 Jeol, Tokyo, Japan). Single-particle scattering spectra were measured with micro-spectroscopy implemented in an AxioImager Z1.m (Carl Zeiss, Jena, Germany) using a tungsten lamp with a color temperature of 3200 K. A spectrometer SpectraPro SP2300i with a camera Pixis 256 (both Princeton Instruments, Trenton, NJ, USA) was coupled via optical fiber to the microscope [30]. The resulting spectra were normalized regarding the light source and the background signal.

### Electromagnetic Simulation

The scattering spectra of the silver particles and the silver particles with the protrusion were calculated by finite element method implemented in a software package Comsol Multiphysics (version 4.3b, RF module). The model of TrNP was defined as a prism with an equidistant triangular base. The edges were rounded by a spherical curvature with a diameter equals to the height of the prism. The dielectric constant of silver was taken from Johnson and Christy [31]. The calculated space was divided into four domains. The first domain contained the TrNP and the second domain the corroded tip. The third domain was a sphere containing the TrNP plus the corroded tip domain with the size of half of the calculated wavelength and refractive index of air. The fourth domain was a perfectly matching layer surrounding the air domain and was used to minimize the reflection of the field. The scattering spectra were calculated by integration of the power flow through a surface of the air (third) domain.

## Results and Discussion

### Observation of Morphological Anisotropy in the TrNPs Degradation Process

**Fig. 1:**
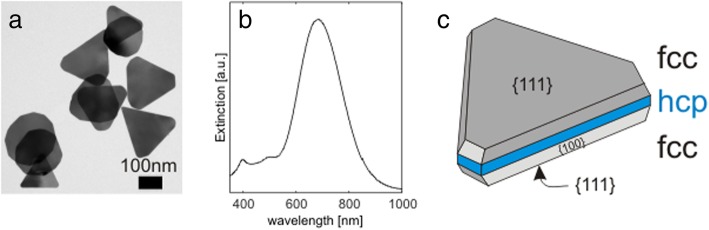
**a** TEM image of the triangular-shaped silver NPs. **b** Extinction spectrum of the corresponding NPs. **c** Schematic of the crystallographic structure of the triangular-shaped Ag nanoparticle

**Fig. 2:**
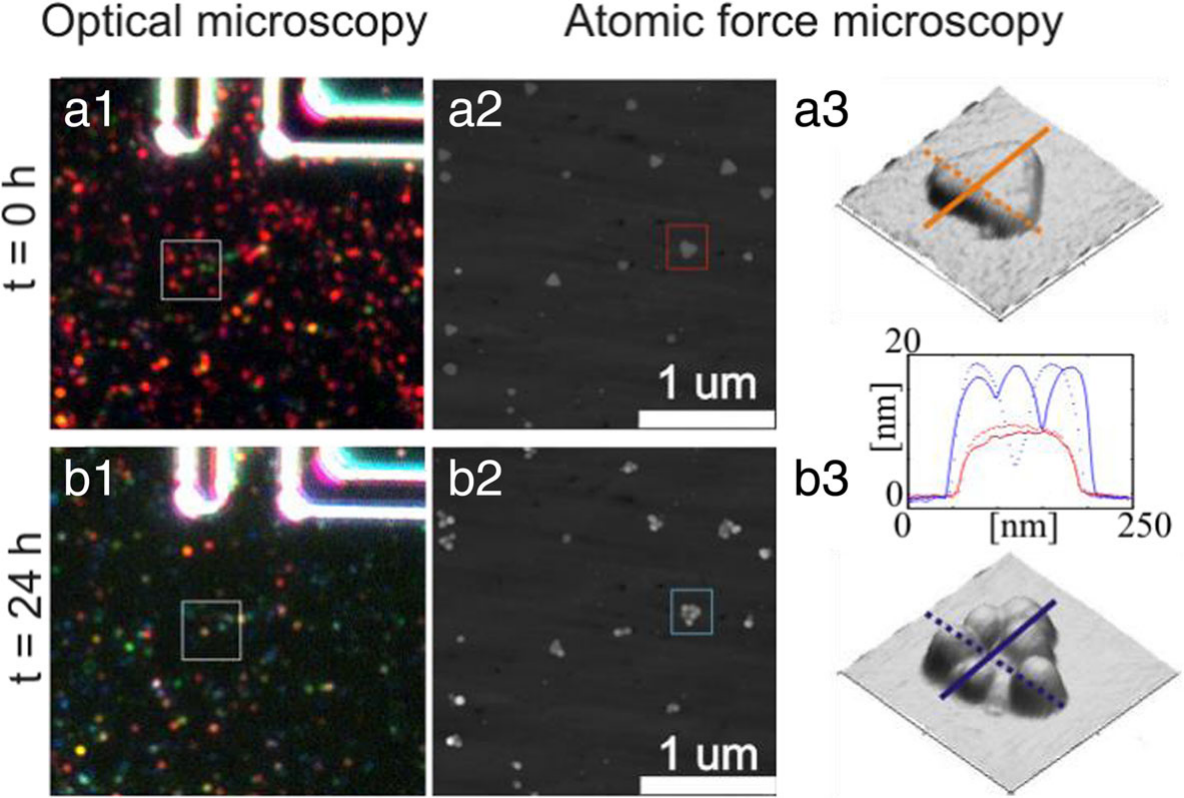
Dark-field images of the same area on a glass substrate with TrNPs (white lines—chromium patterns for particle localization) immediately after deposition (**a1**) and 24 h after deposition left under the standard laboratory condition on air (**b1**). The white squares indicate areas of the recorded topology AFM images immediately after deposition (**a2**) and 24 h after deposition (**b2**). Zoomed 3D topology images of a single particle (indicated by squares in **a2** and **b2**) immediately after deposition (**a3**) and 24 h after deposition (**b3**). The lines indicate the position of profile line displayed in the graph between the 3D images

Silver equilateral triangular-shaped monocrystalline nanoparticles (TrNP) were successfully chemically synthesized in solution. The average side of the triangle was 100 nm and an average thickness of 8 nm. A representative TEM image of TrNPs is in Fig. 1a. The TrNP solution had blue color, and its extinction spectra exhibited strong peak around 710 nm (see Fig. 1b). This peak originates from the dipole plasmon mode lying in plane of the TrNP [32]. The particles were immobilized on a glass substrate in low density in order to resolve the TrNP in an optical microscope with a dark-field illumination setup. An image of such a sample is shown in Fig. 2a1. The deposited single TrNPs are visible as red dots (diffraction-limited spots) in the image. The red color corresponds to the scattered light of the plasmon in the TrNPs (~ 710 nm). Because the spectral position of the plasmon peak is quite sensitive to small changes in the shape of the particle or a presence of neighboring particle, there is some variation in the color and intensity of the spots. Concurrently, a small area of the sample was recorded by AFM (indicated by a white square in Fig. 2a1) as presented in Fig. 2a2. The AFM image confirms that most particles have triangular shape and are sparsely (not aggregated) deposited on the surface. 3D image of one exemplary TrNP (red square in Fig. 2a2) is presented in Fig. 2a3, and it shows that the TrNP is flat with a height of 8 nm (orange profile lines in graph below the image).

The probe was then left for 24 h under normal laboratory condition on air. Afterwards, the identical areas as before were investigated by optical microscopy and atomic force microscopy for its stability. The dark-field images (see Fig. 2b1) immediately showed the alteration of the TrNPs. The intensity of the scattering spots strongly decreased (or completely diminished), and the color changed from red to green/blue. A very interesting insight into the mechanism of the degradation of the TrNP reveals the recorded topological AFM image (see Fig. 2b2). The particles are still present on the substrate, but they undergone distinct morphological changes. The initial triangular-shaped form is still visible, but exhibits extra protrusions, which are laterally confined (~ 50 nm) and located only on the TrNPs’ tips and edges. These protrusions extend substantially over the flat surface of the TrNPs (~ 20 nm, compare blue and orange profile line in Fig. 2b3), and so, they are easily seen in the AFM topology image.

### Time-Resolved Measurement of the TrNPs’ Degradation

**Fig. 3:**
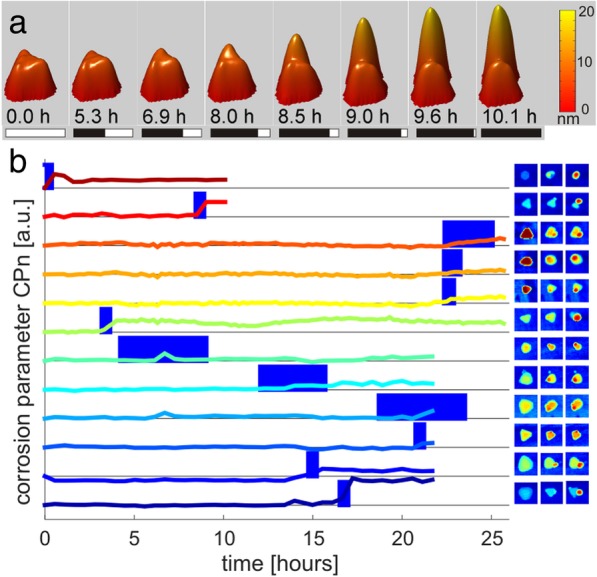
**a** 3D representation of AFM images of a single triangular-shaped silver nanoparticle during the corrosion (red/second from top curve in section **b**) The bar below the image represents the time or recording relative to total time (10.1 h). **b** Graph of the time evolution of the NPs’ corrosion for twelve different NP from three different samples (the lines are offset by 1 for better visibility). The blue bars estimate the major (75%) time of the corrosion. Inset—AFM images of the NP at the beginning, during the strongest corrosion (middle of the blue bar in the graph), and at the end of the measurement

Time-resolved study of the topological changes in single TrNPs was carried out in order to get better insight into the mechanism of the particle corrosion. In the experiment, TrNPs were deposited on the chips and then the sample was AFM scanned in continuous mode for several hours in a specific area of the surface having several immobilized TrNPs. Due to the slow process of the AFM data acquisition with a high spatial resolution, the time resolution was limited to approximately 30 min. A recorded example of the time sequence of the corrosion of the TrNP is presented in Fig. 3a. The figure shows 3D representation of TrNPs at different times from 0 to 10.1 h. At the beginning, the TrNP exhibits regular triangular shape with a flat top surface and an average height of ~ 8 nm. In the first 8 h, there is no visible sign of any morphological changes. It is followed by around 1 h period of a strong growth of a protrusion at the tip of the triangle, which reaches around 20 nm in height. Afterwards, no more changes in the morphology are observed.

In order to be able to compare the corrosion process between different TrNPs, the process had to be quantified from the AFM images by defining a corrosion parameter reflecting the corrosion process. Although the corrosion is characteristic with the growth of the protrusion from the tip, the topological changes are not identical (see Fig. 2b2). Unfortunately, too large noise imposed on the long-time acquisition of AFM data as well as imaging artifacts induced by the finite size of the AFM tip avoided a definition of the corrosion parameter as the relative increase of the volume of the TrNPs. Instead, the flatness of the TrNPs and the effect of horizontal growth of the protrusion were used. We defined the corrosion parameters CPn(*t*) = CP(*t*)/CP(0), CP(*t*) = max(height of TrNP)(*t*)/average(height of TrNP)(*t*), where *t* is time of the recording. For an ideally flat particle (not only restricted triangular shaped), CP(*t*) parameter is equal one. The growth of a spatially localized protrusion causes an increase of the CP(*t*) parameter. Because of the AFM inherent imaging artefacts (tip convolution, noise, etc.), the CP(*t*) parameter is not equal one and varies slightly from one particle to another; the corrosion parameter CPn(*t*) is normalized to one for the beginning of the measurement. We are aware of the fact that this definition is not completely general, but it allows us to quantify the corrosion process in our case.

Three independent time-resolved AFM measurements on different samples were carried out. The scanning size was restricted to only a few micrometers in order to get high resolution of the TrNP topology, and therefore, only few particles were measured. Additionally, a slow spatial drift in the AFM scanning over the long time also reduced the scanning area for later processing. Therefore, from these measurements, the corrosion of twelve TrNPs was followed and their corrosion parameter CPn(*t*) over time is plotted in Fig. 3b. All these curves follow the similar trends as the process observed in Fig. 3a (constant phase, increase of the signal, constant phase). Therefore, a sigmoidal fit was applied on these curves (see Additional file 1: Figure S1). From the fit, the starting time of the corrosion and the time period of the strongest corrosion were defined. They are represented as blue bars in each curve in Fig. 3b (values also in Additional file 1: Figure S1b,c). The data show that in most particles, the corrosion acts less than 1 h, but also longer corrosion occurs. Further, the starting point of the corrosion appears stochastic. AFM images next to the curves represent the TrNP at the beginning of the measurement, at the time of strong corrosion, and at the end of the measurement. The growth of the protrusion from the tip of the TrNPs is visible in these images. The corrosion parameter was also calculated for a not altered TrNP in each measurement in order to check the validity of the definition of CPn(*t*). The curves are presented in Additional file 1: Figure S2, and the AFM images in the inset show that there was not a growth of the protrusions in these particles. The CPn(*t*) curves are constant, which validates the definition of the corrosion parameter.

The observations of the tip-specific corrosion process of the TrNPs can be explained by the model of the crystallographic structure of the particle. According to Aherne et al. [26], a triangular silver nanoparticle consists of 3 layers stacked parallel to the large side of the particle (see image in Fig. 1c). The top and bottom layers are low-defect fcc (face-centered cubic structure) layers with {111} orientation on the top and bottom plane of the triangle. Due to the strong presence of defects in the middle layer, the crystallographic structure of this layer is rearranged to hexagonal closed packed (hcp). This model is used to explain the 2D anisotropic formation of the triangles in the solution. Because the hcp layer is less stable than fcc layer (fcc is the natural crystalline structure of silver), the growth (addition of silver atoms) is much faster at the edges of the hcp structure rather than at the {111} and {100} faces in the fcc layer. There is also other model of Ag TrNP [33], without the hcp layer, trying to explain the particle formation. However, in our study, the presence of the defect-rich hcp layer can be nicely used to explain the observed morphological changes during the corrosion. The particle is stable for certain time till corrosion process is initiated (stochastic process). The corrosion starts with the highest probability from the defects in the hcp layers, which is only accessible from the side of the particles. Because the hpc layer is the most accessible from the triangle tips, the corrosion starts there. During the strong phase of the corrosion, the silver atoms from the hcp layer are mostly involved and the protrusion grows isotopically (as a sphere) with increasing its volume. The growth ends up, when the silver atom in the hcp layer cannot any more efficiently diffuse to the position of the protrusion. Similar effect of hollow triangular particles was observed by a galvanic replacement of silver by gold [34].

Some research groups investigated corrosion of silver nanomaterial such as chemically synthetized nanowires [35], silver triangles [36], and e-beam lithography fabricated nanorods [37]. All these studies were focused on corrosion processes in much longer time spans (over several days/weeks) but with much lower sampling frequency than in our case. They used electron microscopy (SEM/TEM) to image the morphological changes in particles, and they observed a formation of ultra-small particles next to the corroded particles. But these electron microscopy techniques do not allow tracking of the real-time corrosion process, because it demands vacuum to operate, and therefore, only snap shots of mostly different samples at different times are done. We suggest that some of the ultra-small particles observed in these publications are initially formed by our documented mechanism of a growth on the tips of the silver particles. And only afterwards, they are transported to the side of the corroded particles. The transport could be initiated, e.g., by capillary forces in the microscopic layer of H_2_0 on the surface of a substrate or even by radiolytic or knock-on damages of highly energetic electron beam passing through the sample during the TEM study [35].

### Optical Spectroscopy of the Corroded TrNPs

**Fig. 4:**
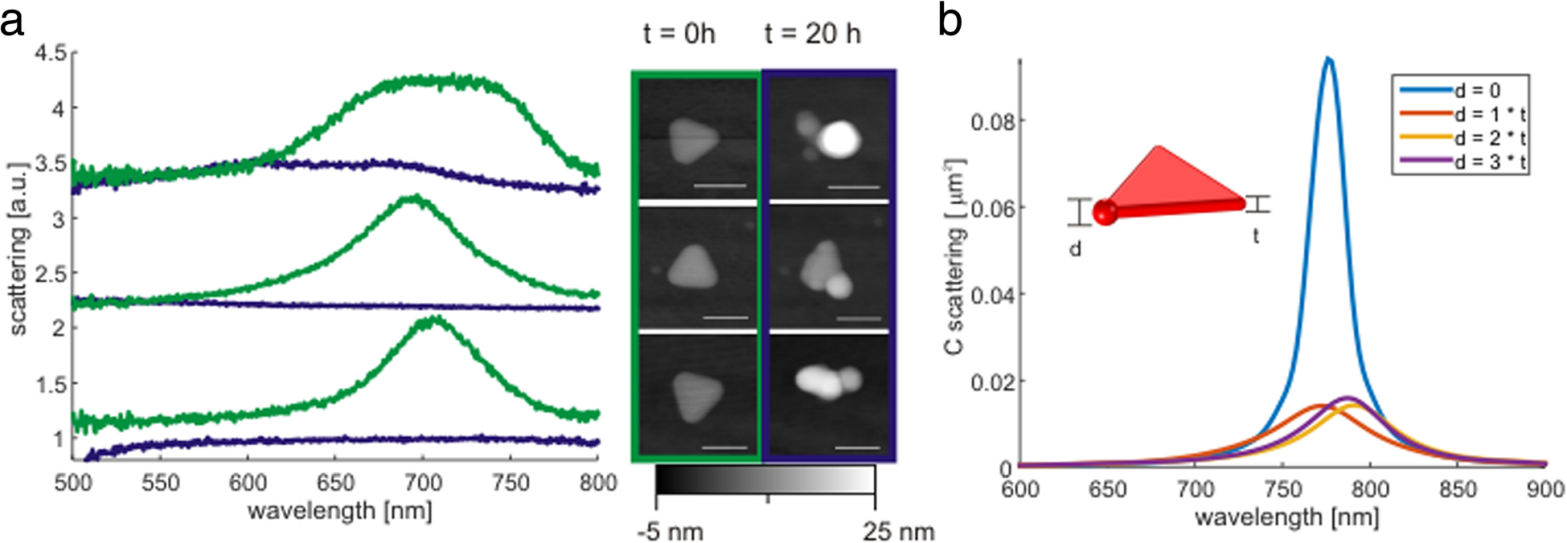
**a** Scattering spectra of three particles before (green) and after (blue) their corrosion. Spectra of each particle were given an offset of 1.1 for better visibility. Inset—their corresponding AFM images before and after corrosion showing grown protrusions. **b** Simulated optical scattering spectra for pure triangular-shaped silver nanoparticle (*d* = 0) and nanoparticle with a protrusion on the tip and with different sizes (*d* = 1, 2, 3 × *t*). Inset shows the calculated geometry of TrNP with the corrosion

The color changes of the TrNPs during the corrosion observed in Fig. 2a and b were studied in more detail by a single-particle micro-spectroscopy. TrNPs were immobilized on a substrate, and their topological AFM images and their scattering spectra were recorded. After 20 h, the AFM topology of the particles and their spectra were recorded again. Three examples of nanoparticles undergoing the corrosion process are displayed in Fig. 4a. At the beginning of the measurement, the TrNPs exhibit nice triangular form with flat top surface as seen in their AFM images and their scattering spectra show specific plasmon peak around 700 nm (green lines). The AFM images of the TrNPs recorded after 20 h indicate that the particle corroded and that protrusions (of different sizes) are grown at their tips. The scattering spectra are completely suppressed (only the top particle still possess very broad weak double peak).

Electromagnetic simulation (based on finite element method [38]) was carried out in order to check if a protrusion placed on the tip of the TrNPs has such a strong influence on the scattering spectra of the particles and what is the influence of the size of the protrusion. First, scattering spectrum of silver TrNPs was calculated and it exhibits the characteristic plasmon peak (see Fig. 4b—blue line). Next, the protrusion was simplified by a sphere and—as an initial guess of the material—the silver sulfide was used (see inset of Fig. 4b). The spectra were calculated for different sphere sizes (1, 2, and 3 times the TrNP height) in order to simulate the growth of the protrusion (plotted in Fig. 4b). The presence of the protrusion leads to strong damping and broadening of the peak even for the smallest size; further increase of the size has only a small impact. This simulation agrees with experimental data. Similar results were achieved when other protrusion materials (for example silver oxide) were assumed. The reason for the strong damping is the highly localized electromagnetic field on the tip of TrNPs and the high absorption of protrusion. Therefore, the simulation supports the idea that the protrusion are made of corroded silver, which leads to strong damping of the plasmon resonance (Ag_2_O, Ag_2_S, Ag_2_CO_3_).

In the atmosphere, typical concentrations of reduced sulfur gases with high sulfidation rates, such as H_2_S, OCS, SO_2_, and CS_2_, are sufficient to initiate the corrosion process [35]. Moreover, the carbonyl sulfide OCS is the principal corroding gas if there is not any sources of hydrogen sulfide H_2_S (OCS + H_2_0 → H_2_S + CO_2_) [39]. Three corrosion mechanisms of silver particles are suggested in the literature [36]: first, direct conversion to Ag_2_S; second, oxidative dissolution of Ag(0) ➔ Ag1+ followed by precipitation as Ag_2_S; and third, oxidative dissolution of Ag(0) ➔ Ag1+ followed by precipitation as Ag nanoparticulate which is terminated by conversion to Ag_2_S. The commonly used techniques (such as X-ray diffraction or extended X-ray absorption fine structure) [40] to determine atomic structure of a crystals require large volumes of a sample, and therefore, they cannot be used for single particle analysis. The transmission electron microcopy (SEM/TEM) allows the detection of the presence of Ag_2_S in a single particle indirectly by analyzing the lattice fringe spacing [36]. Alternatively, energy-dispersive X-ray spectroscopy (EDS) allows elemental analysis of a sample [35]. Nevertheless, our samples (nanoparticles immobilized on glass chips) are not suitable for the investigation by electron microscopy due to the charging of the glass substrate. To avoid this, we covered the glass chip with a thin layer of conduction polymer (Clevios P), prior to deposition of silver particles. The XDS analysis—carried out on clusters of nanoparticles in order to get sufficient signal—did not confirm the presence of sulfur (data not shown). However, we contemplate that the corrosion process is altered—did not observe the characteristic protrusion on the particles in SEM images. It could be that the surface properties influence the corrosion (e.g., by changing the presence of microscopic layer of H_2_0 on the surface of substrate [36] due to the changes in the hydrophilicity of the substrate). Therefore, in our experimental conditions, we could not determine the composition of the observed protrusion and which type of the corrosion mechanism occurs.

## Conclusion

This study showed on a single-particle level that triangular-shaped monocrystalline silver particles corrode on air in highly anisotropic manner. The corrosion is a discontinuous process with a period of quick protrusion growth from the tips of the triangular-shaped particles. The growth of the protrusion is associated with the immediate loss of the plasmon resonance in these particles, which is caused by the strong damping of the localized electromagnetic field in the protrusion. This behavior can be explained by the crystallographic model of the triangular particle consisting of a defect-rich hcp layer sandwiched between more stable fcc crystallographic layers. These new observation can be used to improve the stability of these particles by covering only the tips of the TrNPs by more stable material such as gold. On the other hand, the result can help to design silver nanoparticles with enhanced catalytic activity.

## Additional File

Additional file 1: This file contains supplementary Figure S1 and Figure S2. (DOCX 149 kb)

## Data Availability

The datasets generated during and/or analyzed during the current study are available from the corresponding author on reasonable request.

## Abbreviations

APTES: 1% pre-hydrolyzed 3-aminopropyl-triethoxysilane
EDS: Energy-dispersive X-ray spectroscopy
fcc: Face-centered cubic structure
hcp: Hexagonal closed packed structure
LSPR: Localized surface plasmon resonances
SEM: Scanning electron microscopy
SERS: Surface-enhanced Raman spectroscopy
TEM: Transmission electron microscopy
TrNPs: Triangular-shaped nanoparticles
