# Supplementary material for: Time-Resolved Study of Site-Specific Corrosion in a Single Crystalline Silver Nanoparticle
Source: Nanoscale Res Lett. 2019 Jul 17;14:240. doi: 10.1186/s11671-019-3077-9 (PMC6637113; doi:10.1186/s11671-019-3077-9)
Supplement: Supplementary file 1 — This file contains supplementary Figure S1 and Figure S2. (DOCX 149 kb) [file 11671_2019_3077_MOESM1_ESM.docx]

**Time-resolved study of site-specific corrosion in a single crystalline silver nanoparticle**

Steffen Trautmann^a*^, André Dathe^a*^, Andrea Csáki^a^, Matthias Thiele^a^, Robert Müller^a^, Wolfgang Fritzsche^a^, Ondrej Stranik^a^†

*^a^ Leibniz Institute of Photonic Technology (IPHT) Jena, Member of the Leibniz Research Alliance - Leibniz Health Technologies, Albert-Einstein-Straße 9, 07745 Jena, Germany.*

* contributed equally

† corresponding author: [ondrej.stranik@leibniz-ipht.de](mailto:ondrej.stranik@leibniz-ipht.de)

Fitting procedure of the curve CPn(t) curves

1. CPn(t) curve was scaled to from -1 to 1.
2. Non-linear fit with sigmoidal curve y(t) = 2/pi*atan(a*(t-b))
   1. b .. Inflection point, a ..slope at the inflection point
   2. due to the limited AFM sampling frequency the lower limit of the parameter a was set to 1
   3. the 75% corrosion time was defined as 2/a*tan(pi/2*0.75)


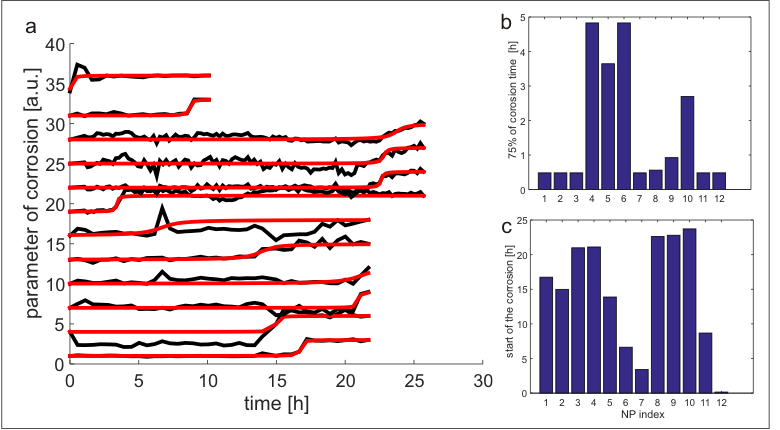


**Figure S1** a- Time evolution of the corrosion parameter (normalized) for each single particle (black line) and their corresponding sigmoidal fit (red line). b – fitted corrosion time (75% of corrosion) for each NP. c – fitted starting point of the corrosion for each NP.


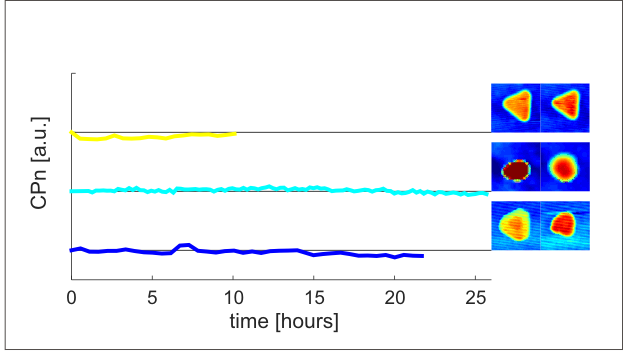


**Figure S2** Graph of the time evolution of the corrosion parameter CPn for an exemplary not altered TrNPs from each measurement set (for better visibility each curve is offset by 1).. Inset – AFM images of the TrNPs at the beginning and at the end of the measurement.
